# Starvation-Survival in Haloarchaea

**DOI:** 10.3390/life5041587

**Published:** 2015-11-12

**Authors:** Yaicha D. Winters, Tim K. Lowenstein, Michael N. Timofeeff

**Affiliations:** Binghamton University, PO Box 6000, Binghamton, NY, USA; yaichawinters@gmail.com (Y.W.); mtimofeeff@yahoo.com (M.T.)

**Keywords:** haloarchaea, algae, *Dunaliella*, starvation-survival, fluid inclusions, halite

## Abstract

Recent studies claiming to revive ancient microorganisms trapped in fluid inclusions in halite have warranted an investigation of long-term microbial persistence. While starvation-survival is widely reported for bacteria, it is less well known for halophilic archaea—microorganisms likely to be trapped in ancient salt crystals. To better understand microbial survival in fluid inclusions in ancient evaporites, laboratory experiments were designed to simulate growth of halophilic archaea under media-rich conditions, complete nutrient deprivation, and a controlled substrate condition (glycerol-rich) and record their responses. Haloarchaea used for this work included *Hbt. salinarum* and isolate DV582A-1 (genus *Haloterrigena*) sub-cultured from 34 kyear Death Valley salt. *Hbt. salinarum* and DV582A-1 reacted to nutrient limitation with morphological and population changes. Starved populations increased and most cells converted from rods to small cocci within 56 days of nutrient deprivation. The exact timing of starvation adaptations and the physical transformations differed between species, populations of the same species, and cells of the same population. This is the first study to report the timing of starvation strategies for *Hbt. salinarum* and DV582A-1. The morphological states in these experiments may allow differentiation between cells trapped with adequate nutrients (represented here by early stages in nutrient-rich media) from cells trapped without nutrients (represented here by experimental starvation) in ancient salt. The hypothesis that glycerol, leaked from *Dunaliella*, provides nutrients for the survival of haloarchaea trapped in fluid inclusions in ancient halite, is also tested. *Hbt. salinarum* and DV582A-1 were exposed to a mixture of lysed and intact *Dunaliella* for 56 days. The ability of these organisms to utilize glycerol from *Dunaliella* cells was assessed by documenting population growth, cell length, and cell morphology. *Hbt. salinarum* and DV582A-1 experienced size reductions and shape transitions from rods to cocci. In the short-term, these trends more closely resembled the response of these organisms to starvation conditions than to nutrient-rich media. Results from this experiment reproduced the physical state of cells (small cocci) in ancient halite where prokaryotes co-exist with single-celled algae. We conclude that glycerol is not the limiting factor in the survival of haloarchaea for thousands of years in fluid inclusions in halite.

## 1. Introduction

Microbial communities living in salt-saturated waters may survive during periods of desiccation trapped in brine inclusions in halite. Some microbes are released when surface salt crusts are dissolved by rare floods [1,2]; others remain trapped in halite crystals to be buried for millennia. How long are cells able to survive inside salt crystals? Norton and Grant [3] reported that 13 different halobacteria remained viable for a minimum of six months. This was determined by their ability to regrow cells from isolated cultures and natural mixed populations that were trapped within laboratory-grown crystals of halite. Other studies suggest halotolerant and halophilic prokaryotes may survive inside fluid inclusions in halite on geologic timescales, some since the Paleozoic. Schubert *et al.* [4] found viable halophilic archaea in 22 ka, 25 ka, and 34 ka halite from Death Valley, CA. Mormile *et al.* [5] cultured *Hbt. salinarum* from 97 ka old halite in Badwater salt pan, Death Valley. Gramain *et al.* [6] cultivated an organism closely related to *Hbt. noricense* from ~1.8 Ma halite from Salar Grande, Chile. Vreeland *et al.* [7,8] isolated a spore-forming bacterium, *Bacillus species 2-9-3*, from a 250 million year old halite crystal from the Permian Salado Formation in New Mexico, and isolated six strains of Cretaceous (121–112 Ma) haloarchaea from halite crystals of the Sergipe Basin, Brazil. Stan-Lotter *et al.* [9] reported the isolation of *Halococcus salifodinae* from Permo-Triassic salt deposits in Austria, Germany, and England.

Successful cultivation of prokaryotes from ancient salt has been viewed with skepticism. Isolates are considered by some to be modern contaminants because of DNA similarity to modern relatives [10,11,12]. Although contamination can occur if halite sterilization procedures are inadequate, if foreign windblown (cyclic) salt enters a sampling site [13], or by salt recrystallization after deformation or dissolution while in contact with younger pore fluids [14], a growing number of reports have shown cultivated isolates with credible ancient ages. There is also much to learn about DNA repair of cells living in a fluid inclusion with little oxygen, the disposal of toxic waste products within a closed system, and sustenance acquisition for cell maintenance and reproduction. Therefore, thorough examination of species cultured from ancient salt are needed to determine if their long-term survivability is plausible.

### 1.1. Haloarchaeal Advantages for Long-Term Survival

Halophiles live in extreme habitats such as concentrated brines in salt pans, subject to contrasting flooded and desiccated conditions. Therefore, it may not be surprising that the most common group of microorganisms isolated from ancient salt has been haloarchaea [4,5,6,8,15,16,17,18,19]. Haloarchaea have several advantages for longevity while trapped in halite. For example, haloarchaea do not form spores, allowing them to remain metabolically active at all times [20,21]. They produce carotenoids when stressed, which fight free radicals that can damage DNA [22,23]. Haloarchaea also concentrate K^+^ ions internally to balance the osmotic pressure of surrounding brines [24]; cellular fluids with high ionic strength slow the rate of DNA depurination [25]. There is evidence that some haloarchaea, e.g., *Haloferax volcanii*, are polyploid so they may use DNA as a source of phosphate if it were not available inside a fluid inclusion [26,27].

Haloarchaea have also adapted to grow with limited oxygen because of its low solubility in brines [14]. In nature, under low oxygen conditions, some haloarchaea, such as *Hbt. salinarum*, synthesize the protein bacteriorhodopsin to obtain cellular energy from sunlight [28,29,30]. This light-mediated ATP production provides energy to haloarchaea [21] living in the water column or to cells trapped in fluid inclusions before burial, while sunlight can still penetrate crystals. When sunlight is unavailable after crystal burial, at least one haloarchaeal species, *Hbt*. *salinarum*, can acquire alternative energy through the fermentation of arginine [31]—beneficial to an organism trapped in a low-oxygen subsurface environment for millennia. These characteristics make halophiles well suited for survival in ancient salt.

### 1.2. Starvation-Survival

If energy-yielding substrates are absent, microbes persist through a process called starvation-survival [32]. Starvation-survival is essential for prokaryotic persistence in natural habitats low in available nutrients, such as ancient fluid inclusions. Researchers have characterized the response of many marine bacterial species to nutrient limitation. These include Cavicchioli *et al.* [33] who studied *Sphingopyxis alaskensis*, Srinivasan and Kjelleberg [34] on a *Vibrio* species, Hood *et al.* [35] on *Vibrio cholerae*, Amy and Morita [36] on sixteen marine bacteria, and Novitsky and Morita [37,38] on a psychrophilic *Vibrio*. Some cells, depending on the species or strain, altered behavior (e.g., chemotactic response, respiration rate, adhesiveness, motility) or physical structure (e.g., size, shape, molecular constituents) in response to the stress experienced. For example, experiments performed with marine species (*Vibrio cholerae*, unidentified isolate S14, psychrophilic *Vibrio* ANT-300 and 16 open-ocean isolates) in nutrient-free solutions revealed a routine transformation from rod-shaped cells to spherically shaped cells after several weeks [35,39,40,41].

Starvation responses are less well known in haloarchaea, organisms frequently sealed in fluid inclusions in halite crystals for long periods of time. Schubert *et al.* [2] observed that nearly all ancient prokaryotes trapped in halite crystals (≥10,000 years old) from the Death Valley salt core were small coccoid-forms (<1 μm in diameter); it was rare to find rod-shaped cells (<2.5 μm long). This contrasted with larger rod-shaped prokaryotes (1–10 μm long) commonly residing with small cocci (1–2 μm in diameter) in relatively young (1 to 26-year-old) fluid inclusions in halite crystals from Saline Valley. Schubert *et al.* [2] concluded that large cells reduced in size, or miniaturized, as nutrients were exhausted in the brine pockets trapped in halite sometime between 26 and 10,000 years ago.

Norton and Grant [3] simulated the nutrient exhaustion experienced in ancient salt by studying how haloarchaeal entrapment in fluid inclusions influenced their survival. Rod-shaped cells became smaller and more spherical after several weeks, similar to the response of many marine species. The physical change was likely due to reductive division or miniaturization (see definitions); however, without an assessment of population size, we cannot be certain. The timeline of cellular transformation observed by Norton and Grant [3] (weeks) contrasted with that of Schubert *et al.* [42] (decades to thousands of years) possibly due to substrate availability. Norton and Grant monitored cell suspensions in crystals made from nutrient-free media whereas Schubert *et al.* observed cells in crystals grown from natural Death Valley brines. Methods used in this study differ from Norton and Grant [3] and Fendrihan *et al.* [43] by: (1) adding a number of cell rinses to remove nutrients and induce starvation conditions immediately; (2) recording average cell length and average population size at regular intervals to determine when each survival strategy begins and ends; and (3) including images to document any visual changes.

More research is needed to better understand the metabolic state of haloarchaea trapped in fluid inclusions in halite for millennia [18], the details of their size reduction, and morphological changes [4]. We expected that the physical transformations in haloarchaea observed by Schubert *et al.* [4] in the Death Valley core and by Norton and Grant [3] in their laboratory-grown crystals were caused by nutrient limitation as part of the starvation-survival process, and that the response should vary by organism. Here we advance the knowledge of long-term survival of haloarchaea in fluid inclusions and the timing of cell miniaturization. Morphological transformations that occur during nutrient-rich growth and starvation of *Hbt*. *salinarum* and DV582A-1 were closely examined. It is important to investigate the timing of morphological transformations in haloarchaea in response to nutrient limitation to understand why long rods are alive in fluid inclusions as old as 26 years, and why nearly all cells in ancient (~30 ka) halite are miniaturized and spherical. This study of responses to short-term starvation provides insight into ancient haloarchaeal survival in halite crystals where nutrients are thought to eventually expire.

### 1.3. Haloarchaea and Dunaliella

Haloarchaea have many shapes including rods, cocci, triangles, squares, and disks, <1–15 microns in size (personal observations). They are primarily heterotrophic, which allows them to consume glycerol, among other organic compounds, for nutrition [44]. Organic carbon is readily available because of the high productivity of many hypersaline environments and the evaporative nature of brines [24]. These substrates are expected to be trapped along with haloarchaea in fluid inclusions. Their populations in saline systems can reach 2 × 10^7^ to 1 × 10^8^ cells/mL [45,46,47,48,49] coincident with *Dunaliella* blooms [44].

Studies by Oren [44] and Oren and Gurevich [47] attempted to establish the use of glycerol as a substrate for haloarchaea. Glycerol added to cultures of *Haloferax* and certain *Haloarcula* species was used to produce substrates, e.g., d-lactate and acetate, and pyruvate and acetate, respectively, from which many haloarchaea can grow [47]. Glycerol is the favored energy source for haloarchaea in the Dead Sea because it increased the respiratory activity of haloarchaea more than other carbon compounds [44]. The relationship between haloarchaea and *Dunaliella* is also reviewed by Stan-Lotter and Fendrihan [19].

Schubert *et al.* [18,42] suggested that glycerol, produced in abundance by the alga *Dunaliella*, may have been responsible for the survival of three haloarchaeal isolates they cultured from Death Valley halite, 22 ka, 25 ka and 34 ka in age. Supporting evidence included: (1) the only halite crystals that yielded growth contained both prokaryotes and algae within fluid inclusions; (2) carotenoids produced by algae leaked out of their cell structures, so it was assumed that glycerol leaked as well; and (3) the three isolates were cultivated with a medium containing glycerol.

**Figure 1:**
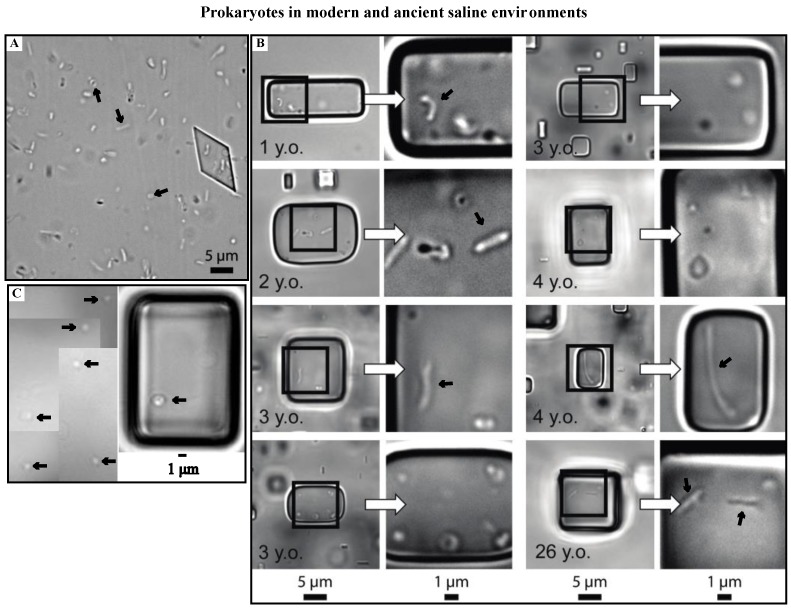
Shows photomicrographs of prokaryotes in brine, modern halite, and ancient halite taken under transmitted light. (**A**,**B**) Modified from Figure 6 and Figure 7, respectively, in Schubert *et al.* [2]; (**A**) Wet mount of prokaryotes in modern brine collected from Saline Valley in March 2004. Black arrows point to various prokaryote morphologies including a disk, rod, and coccus (L → R); (**B**) Prokaryotes in fluid inclusions in Saline Valley halite 1–26 years old. Black arrows highlight rods (>2 µm in length) found in these inclusions; (**C**) Spherically-shaped prokaryotes (cocci) trapped in fluid inclusions in ancient Saline Valley halite. Sample was collected from sediment core SV-4A at a depth of 18.3 m. Black arrows point to cocci.

Prior to culturing three isolates, Schubert *et al.* [2] carefully examined prokaryotes in fluid inclusions from ancient halite crystals, Death Valley. Prokaryotes from modern brines (Figure 1A,B) [3,42,43] were larger, often rod-shaped, and >1 micron in diameter, compared to cells from Death Valley halite (Figure 1C) [2,42]. The dwarfing of ancient cells trapped in fluid inclusions within halite crystals has been attributed to starvation [2,3,43], but the timing of this process in not well known. Schubert *et al.* [2] observed that prokaryotes trapped in fluid inclusions in modern halite collected since 1980 resemble the morphology of cells in modern brines (Figure 1B), whereas prokaryotes in ancient halite (10–25 ka) are much smaller (Figure 1C); they concluded that dwarfing occurred in these cells over periods between 26 years and 10 kyear. Laboratory experiments show that dwarfing of haloarchaea can happen much faster. For instance, Norton and Grant [3] witnessed numerous genera of haloarchaea getting smaller within two to three weeks of entrapment in nutrient-free fluid inclusions. The dwarfing timeline observed by Norton and Grant was similar to that of many marine prokaryotes undergoing starvation [36,37,38,50].

The purpose of this research was to document (1) starvation survival; and (2) test the hypothesis that glycerol, a carbon source for haloarchaea, might enable these organisms to survive for millennia in fluid inclusions. This was examined by exposing otherwise starved haloarchaea, *Hbt. salinarum* and DV582A-1, to a mixture of lysed and intact *Dunaliella* cells and subsequently monitoring their physical responses.

## 2. Results

Population dynamics and morphological transformations of *Hbt*. *salinarum* and DV582A-1 were first recorded in response to nutrient-rich media. Substrates were not replenished in nutrient-rich media after inoculation. The same characteristics were monitored in nutrient deprived cells, and then compared. A study of their responses to short-term starvation provides insight to ancient haloarchaeal survival in halite crystals where nutrients eventually deplete.

### 2.1. Response to Nutrient-Rich Media—Hbt. salinarum

**Figure 2:**
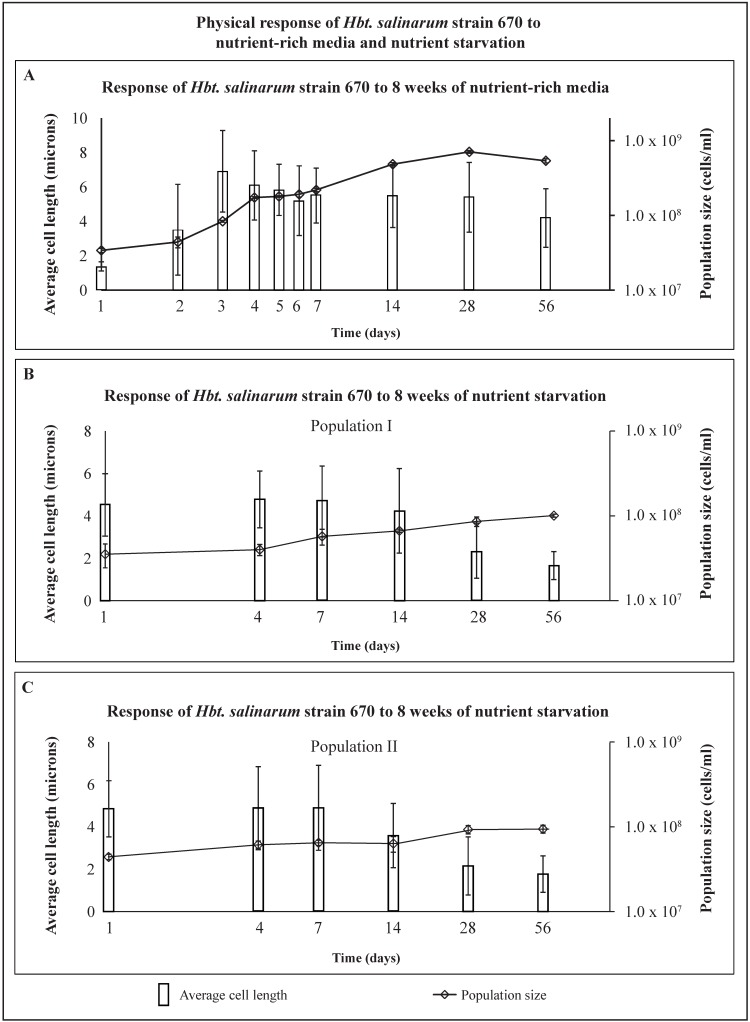
Shows the response of *Hbt. salinarum* strain 670 to (**A**) nutrient-rich media; (**B**) nutrient starvation in population I; and (**C**) nutrient starvation in population II over 56 days of experiments. Average cell length (microns), left axis, shown as vertical bars with standard deviation in brackets. Population size (cells/mL), right axis, is shown as small diamonds with standard deviation in brackets.

**Figure 3:**
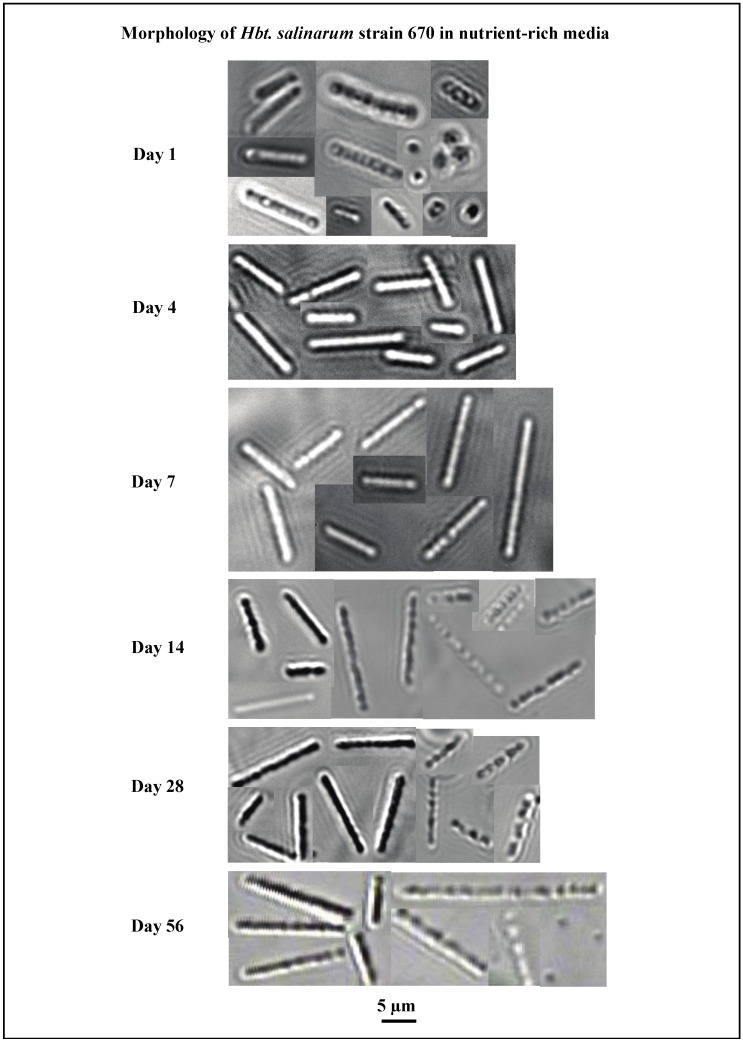
Morphological characteristics of *Hbt. salinarum* strain 670 in nutrient-rich media over 56 days. Panels show photos of cells captured on days 1, 4, 7, 14, 28, and 56. Most cells were rod-shaped throughout the experiment. At two weeks, a large number of rods began to have chain-like appearances, likely due to gas vesicles. Some chains began to dissociate at 56 days. Photographs of DV582A-1 are not presented because the physical changes strongly resembled those of *Hbt. salinarum* shown here.

Population size and cellular lengths of *Hbt*. *salinarum* were measured on days 1–7, 14, 28, and 56 in nutrient-rich media (Figure 2A). *Hbt. salinarum* exhibited a biphasic growth pattern. Average initial cell length was 1.4 ± 0.3 μm (day 1) and peaked at 6.9 ± 2.4 μm on day 3 (Figure 2A). Between day 3 and day 56, the average cell length dropped to 4.2 ± 1.7 μm. However, this decrease was within the standard deviation. Cell density grew from 3.4 × 10^7^ (±2 × 10^6^) cells/mL on day 1 to its maximum value of 7.1 × 10^8^ (±3 × 10^7^) cells/mL on day 28, and then decreased to 5.4 × 10^8^ (±3.4 × 10^7^) cells/mL by day 56 (Figure 2A). The rod-shape of cells was retained throughout the experimental period (Figure 3, day 56).

### 2.2. Response to Experimental Starvation—Hbt. salinarum

**Figure 4:**
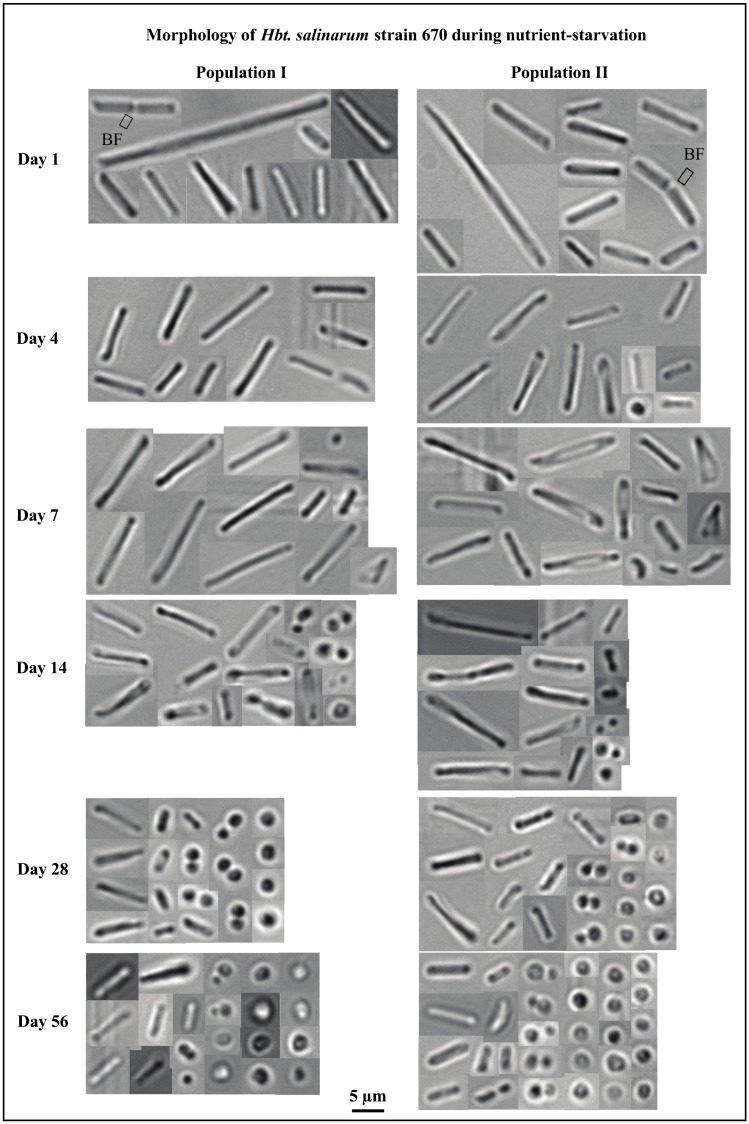
Morphological changes in two populations of *Hbt. salinarum* strain 670 during 56 days of starvation. Rod-shaped cells dominated the populations at the start of starvation, but pleomorphic properties emerged as starvation progressed. Photographs of DV582A-1 are not shown here. DV582A-1 was less pleomorphic than *Hbt. salinarum*, and does not exhibit triangular or safety pin-shaped cells. The physical change from rods to cocci greatly resembled that of *Hbt. salinarum* strain 670. Binary fission is abbreviated as “BF”.

Cells were harvested from nutrient-rich media in late exponential/early stationary phase (Figure 3, day 4) for experimental starvation. The physical behavior of these cells, washed of all nutrients on day 1 (Figure 2B,C), was compared with cells in an aging nutrient-rich media culture (Figure 2A, starting on day 4). Two populations of starved cells were tested to verify that starvation processes were reproducible (Figure 2B,C). Starved cells were pleomorphic (Figure 4). Rod-shaped cells (day 1) were quickly joined by cocci (populations I and II, days 7–56), as well as triangular (populations I and II, day 7) and “safety-pin” shaped cells (population I, day 14, and population II, day 7). Cocci dominated the starved populations by day 28 as rods, triangles, and safety pins became less common.

Both populations of starved cells had statistically significant growth between days 1 and 56. Starved population I increased from 3.5 × 10^7^ (±1.1 × 10^7^) cells/mL to 1.0 × 10^8^ (±4.2 × 10^6^) cells/mL (Figure 2B) and starved population II increased from 4.4 × 10^7^ (±3.5 × 10^6^) cells/mL to 9.4 × 10^7^ (±1.0 × 10^7^) cells/mL (Figure 2C). Average cell lengths declined over the same period. The average cell length of starved population I decreased from 4.5 ± 1.5 μm (day 1) to 1.7 ± 0.7 μm (day 56). The average cell length of starved population II decreased from 4.9 ± 1.3 μm (day 1) to 1.8 ± 0.9 μm (day 56). Growth monitoring was terminated after 56 days, when average cell size approached 2 μm and most cells had coccoidal morphologies (Figure 4).

Reductive division [51] or fragmentation [52,53,54] may have been responsible for growth in both starved populations after week 1. It is important to note that reductive division is not caused by starvation [51]. Reductive division occurs if a cell replicates its DNA when nutrients are available during the growth process, but divides during stationary phase. This type of growth increases population size; however cells do not gain biomass. Reductively divided cells are often coccoid-shaped [51]. For example, starved population I went through reductive division as it grew from 5.7 × 10^7^ (±1.2 × 10^7^) cells/mL (day 7) to 1.0 × 10^8^ (±4.2 × 10^6^) cells/mL (day 56) and average cell size dropped from 4.7 ± 1.7 μm to 1.7 ± 0.7 μm (Figure 2B and Figure 3, population I). Starved population II increased from 6.5 × 10^7^ (±4.6 × 10^6^) cells/mL (day 7) to 9.4 × 10^7^ (±1 × 10^7^) cells/mL (day 56), as the average cell size decreased from 4.9 ± 2 μm to 1.8 ± 0.9 μm (Figure 2C and Figure 3, population II).

After 56 days, the average cell length remained substantially larger in the nutrient-rich media population (4.2 μm) (Figure 2A and Figure 3) than the average cell length in starved population I (1.7 μm) (Figure 2B and Figure 4) or starved population II (1.8 μm) (Figure 2C and Figure 4). Most cells grown in nutrient-rich media were rod-shaped after 56 days but many transformed into chain-like rods (Figure 3, days 14–56). Nearly all starved cells changed from long to short rods and miniaturized cocci after 56 days of starvation (Figure 4).

### 2.3. Response to Dunaliella—Hbt. salinarum

**Figure 5:**
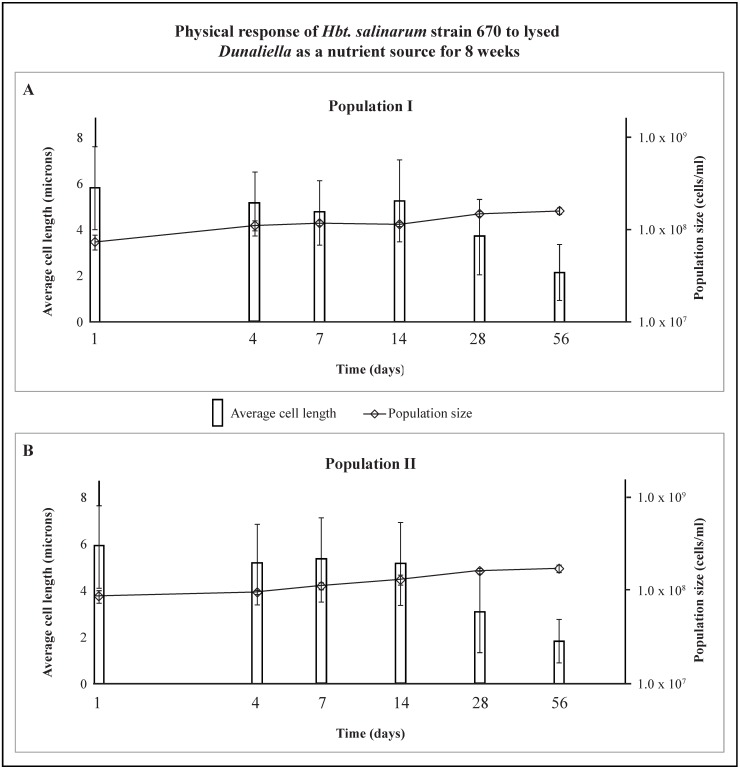
Shows the response of two nutrient-free populations of *Hbt. salinarum* strain 670 exposed to lysed *Dunaliella* over 56 days. Average cell length (microns), left axis, is shown as vertical bars with standard deviation in brackets, and population size (cells/mL), right axis, is shown as small diamonds with standard deviation in brackets. Average population sizes increased as average cell lengths decreased in both populations.

When starved cells of *Hbt. salinarum* (population I) were introduced to a mixture of lysed and intact *Dunaliella* (day 1), cells had an average length of 5.8 µm (Figure 5A). Cell lengths fluctuated until day 14 after which they decreased from 5.3 µm to 2.1 µm (day 56) (Figure 5A). The average population size gradually rose from 7.3 × 10^7^ cells/mL (day 1) to 1.6 × 10^8^ cells/mL (day 56). Starved cells of *Hbt. salinarum* (population II) followed the same trend as population I. Average cell length was 5.9 µm on day 1 and varied slightly before day 14 at which point cell lengths decreased until day 56 (Figure 5B). The average cell length fell from 5.1 µm on day 14 to 3.1 µm on day 28, and then to 1.8 µm on day 56 when the monitoring concluded (Figure 5B). Similar to population I, the average size of population II gradually increased from 8.7 × 10^7^ cells/mL (day 1) to 1.7 × 10^8^ cells/mL (day 56), (Figure 5B).

**Figure 6:**
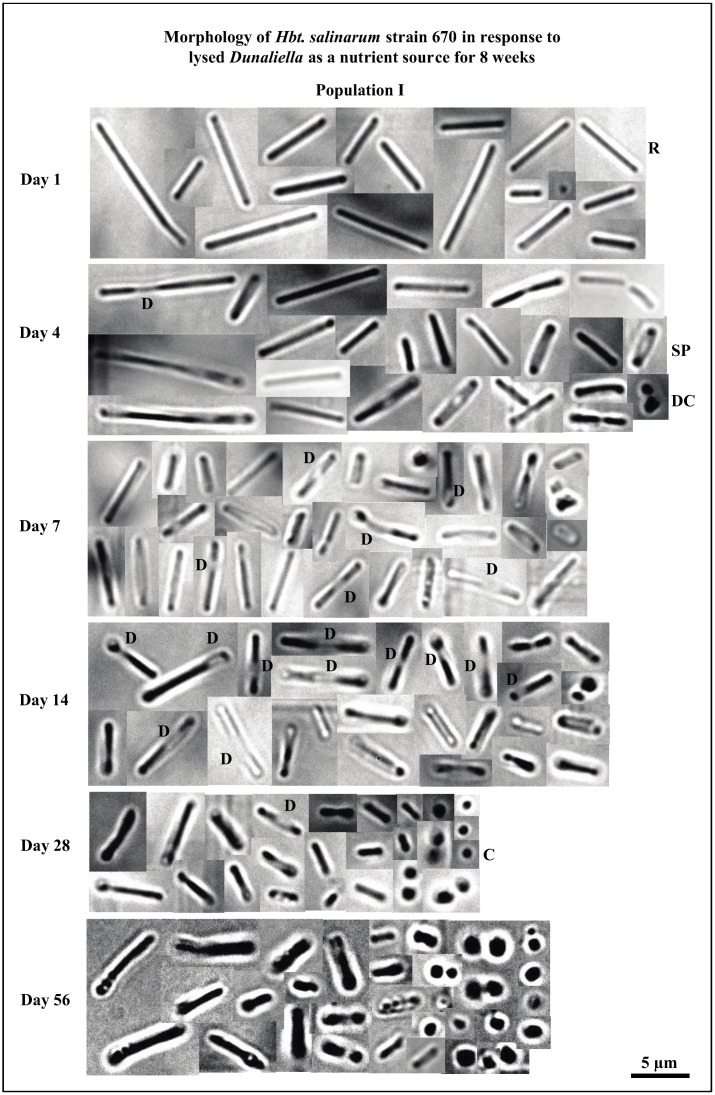
Shows the morphology of *Hbt. salinarum* strain 670 population I when exposed to lysed *Dunaliella* in otherwise sterile brine for 56 days. Population I was dominated by long rods until day 4, after which rods shortened and the number of cocci and diplococci increased. Cocci became the most abundant cell shape within 56 days. Abbreviations: C—cocci; D—degraded/damaged cell structure; DC—diplococci; R—rod; SP—safety pin.

The morphology of *Hbt. salinarum* populations I and II in the presence of *Dunaliella*, showed a similar progression over the 8 week period. Long slender rods dominated the populations until day 4 (Figure 6). Rods commonly appeared damaged after day 4; their outer structures look intact, but there were missing or deteriorated sections in many cells (days 4–14 represented by “D”, Figure 6). Spherical cells (cocci) were more common, beginning on day 28. By day 56, the number of rods declined greatly; cocci and diplococci became the dominant cell shape (Figure 6).

### 2.4. Response to Nutrient-Rich Media—DV582A-1

**Figure 7:**
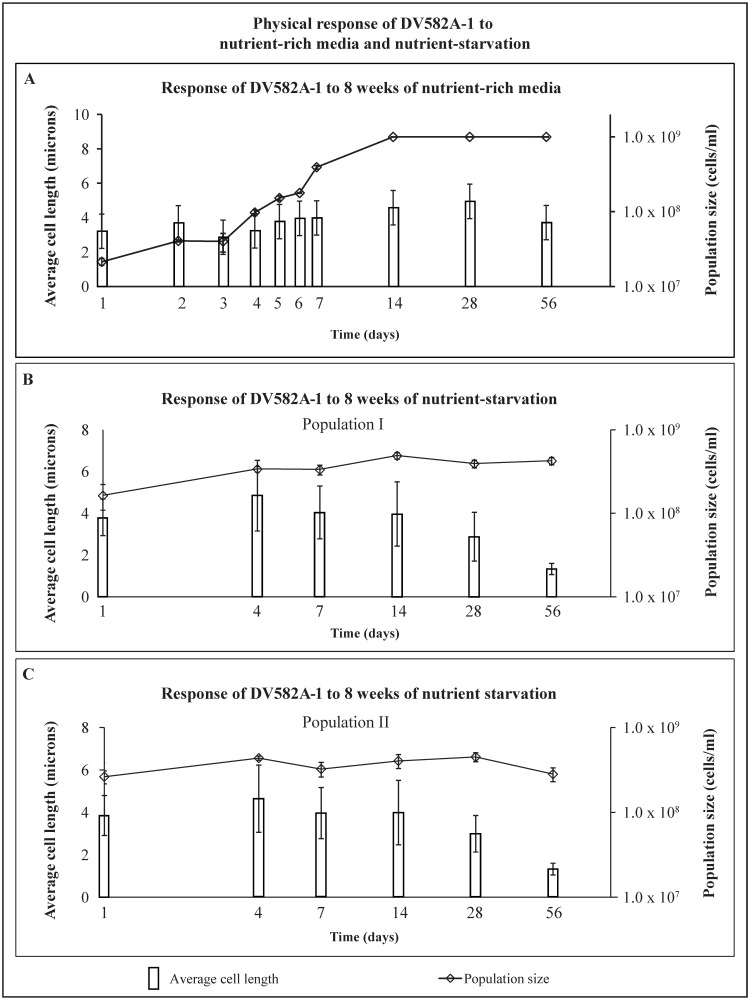
Shows the response of DV582A-1 to (**A**) nutrient-rich media; (**B**) nutrient starvation in population I; and (**C**) nutrient starvation in population II over 56 days. Average cell length (microns), left axis, is shown as vertical bars with standard deviation in brackets, and population size (cells/mL), right axis, is shown as small diamonds with standard deviation in brackets.

DV582A-1 exhibited monophasic growth (Figure 7A). DV582A-1 grew from day 1 until at least day 28; the population increased from 2.1 × 10^7^ (±2.3 × 10^6^) cells/mL with an average cell size of 3.2 ± 0.9 μm to 1.0 × 10^9^ cells/mL and 4.9 ± 1.9 μm, respectively. The culture remained in stationary phase on day 56; there was no indication of population decline. Average cell length fell slightly from 4.9 ± 1.9 μm (day 28) to 3.7 ± 1.4 μm (day 56), but was not statistically significant. DV582A-1 was rod-shaped throughout the experiment.

### 2.5. Response to Experimental Starvation—DV582A-1

Cells were harvested from nutrient-rich media in the exponential phase (day 4) for experimental starvation. The physical behavior of these cells, washed of all nutrients on day 1 (Figure 7B,C), was compared with cells in an aging nutrient-rich media culture (Figure 7A, starting on day 4). Starved populations I and II of DV582A-1 varied slightly in their response. DV582A-1 started the starvation period as rod-shaped. The density of population I increased from 1.6 × 10^8^ (±5.5 × 10^7^) cells/mL (day 1) to 4.2 × 10^8^ (±4.2 × 10^7^) cells/mL (day 56) and the average cell size dropped from 3.8 ± 0.9 μm to 1.3 ± 0.3 μm (Figure 7B). Population II increased from 2.6 × 10^8^ (±4.6 × 10^7^) cells/mL (day 1) to 2.8 × 10^8^ (±5.2 × 10^7^) cells/mL (day 56), but was not statistically significant (Figure 7C). The average size of a cell in starved population II decreased from 3.8 ± 0.9 μm (day 1) to 1.3 ± 0.3 μm (day 56).

The number of starved cells and the average cell lengths declined in population I after day 14 and in population II after day 28 for the remainder of the experimental period. This was the result of miniaturization, which occurs when reductive division has completed. The average cell length of population I decreased between days 14 and 56, from 4.0 ± 1.5 μm to 1.3 ± 0.3 μm. Cells also shortened in population II; the average cell length decreased from 3.0 ± 0.9 μm on day 28 to 1.3 ± 0.3 μm on day 56. By the conclusion of the experiment, DV582A-1 had transformed from long rods to short rounded rods or cocci (data not shown).

### 2.6. Response to Dunaliella—DV582A-1

**Figure 8:**
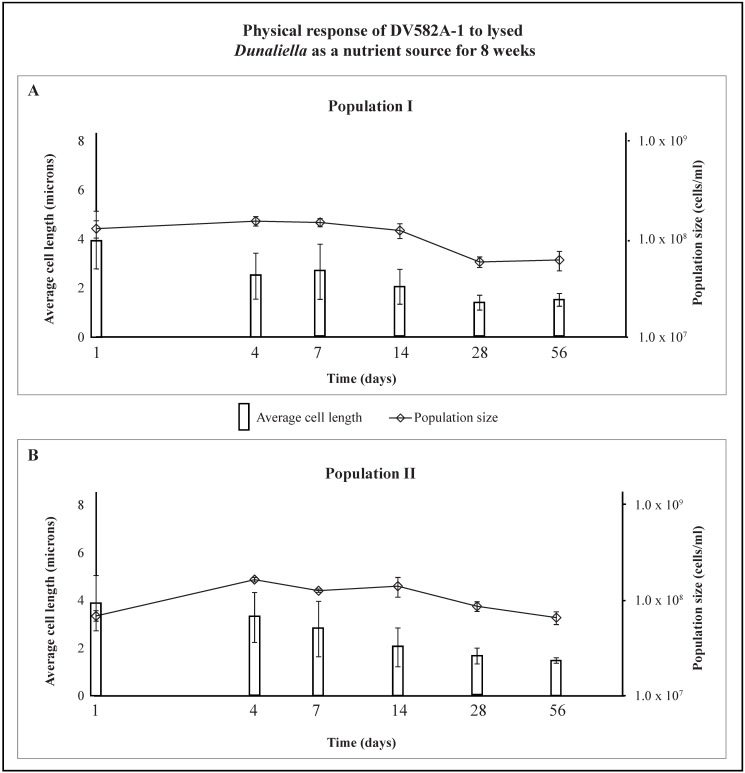
Shows the response of two nutrient-free populations of DV582A-1 exposed to lysed *Dunaliella* in otherwise sterile brine over 56 days. Average cell length (microns), left axis, is shown as vertical bars with standard deviation in brackets, and population size (cells/mL), right axis, is shown as small diamonds with standard deviation in brackets. In both populations, the average population size increased until day 4, then decreased; average cell lengths decreased over the experimental period.

**Figure 9:**
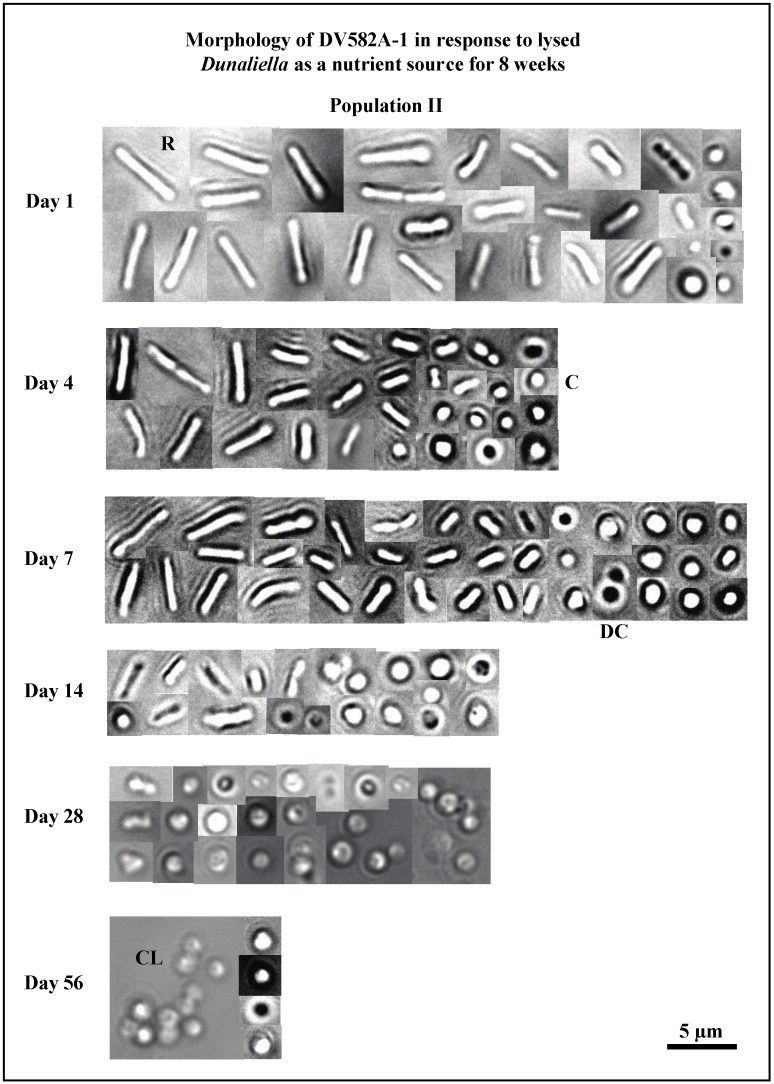
Shows the morphology of DV582A-1 (population II) when exposed to lysed *Dunaliella* in otherwise sterile brine for 56 days. Long rods are abundant on day 1. Subsequently, rods shorten and the number of cocci increase. Clusters of spherical cells appear on days 28 and 56. Cocci are the dominant cell form by day 56. Abbreviations: C—cocci; CL—cluster of cocci; DC—diplococci; R—rod.

Initially, populations of DV582A-1 had smaller average cell lengths (3.9 µm, Figure 8) than *Hbt. salinarum* (5.8–5.9 µm, Figure 3). The average cell length of DV582A-1 (population I) declined to 2.0 µm by day 14 (Figure 8A). The size of population I was unchanged for the first 14 days (Figure 8A). Between days 14 and 28, the population decreased from 1.2 × 10^8^ cells/mL to 5.8 × 10^7^ cells/mL. The population size stayed lower (6.1 × 10^7^ cells/mL) until the experiment ended. The average cell size of population II decreased from 3.9 µm (day 1), 2.0 µm (day 14), and 1.5 µm (day 56) (Figure 8B). Unlike population I, the average size of population II increased between day 1 (6.8 × 10^7^ cells/mL) and day 4 (1.6 × 10^8^ cells/mL), after which it declined to 6.5 × 10^7^ cells/mL (day 56) (Figure 8B). Cells of DV582A-1 were primarily rods at the start of the experiment (Figure 9, day 1). There was a gradual increase in the number of cocci with time. Clusters of spherical cells were present on days 28 and 56.

## 3. Discussion

### 3.1. Summary of Response to Nutrient-Rich Media Versus Experimental Starvation—Hbt. salinarum and DV582A-1

*Hbt. salinarum* reacted differently when suspended in a nutrient-rich solution and a nutrient-free solution. Cells in nutrient-rich media for 56 days were larger than nutrient-depleted cells (4.2 µm *versus* 1.8 µm). Cells used to inoculate nutrient-rich media were initially spherical and rod-shaped (day 1), after which, rod-shaped cells of various lengths dominated the population. Some rods exhibited a chain-like appearance, possibly from gas vesicles or from rapid cell division (before separation), starting on day 14. Gas vesicles can be produced by many haloarchaea, including *Halobacterium* NRC-1, to allow vertical flotation to a more oxygen-rich region [55,56]. Cells of *Hbt. salinarum* experiencing nutrient deprivation appeared dramatically different from cells in nutrient-rich media. Starved cells were more diverse (rods, cocci, triangles, safety-pins, and diplococci) than cells in nutrient-rich media. At the conclusion of the experiments, most starved cells were cocci. In addition to the physical differences observed between cells grown in nutrient-rich *versus* starved conditions, there were large differences in the number of cells produced in each population. The population with nutrients available concluded the experiment with more than five times the number of cells than the starved populations.

Similar results on physical characteristics and population size were obtained for experiments with DV582A-1. Cells in nutrient-rich media over 56 days were larger than the starved populations (3.7 μm *versus* 1.3 μm). Rods were the most abundant cell shape in nutrient-rich media. In starved populations, rods became fewer in number as they converted to cocci. Starved populations of DV582A-1 displayed less variety in cell shapes than *Hbt. salinarum*; triangle and safety pin shapes were absent. The population size in nutrient-rich media was 2 to 3 times larger than the starved populations.

### 3.2. Summary of Growth Strategies in Nutrient-Rich Media

*Hbt. salinarum* and DV582A-1 are rod-shaped haloarchaea of similar length and salt preference [42,57]. When nutrients are available, both organisms replicate using binary fission and each daughter cell grows to the size of the parent cell. The average cell length was greatest for *Hbt. salinarum* in nutrient-rich media on day 3 whereas maximum cell length for DV582A-1 was on day 28.

### 3.3. Variations in Starvation Response

Most cells of *Hbt. salinarum* and DV582A-1 became smaller and rounded in response to starvation conditions, but some cells of the same species and the same population remained rod-shaped and above average in length. This is consistent with the observations of Schubert *et al.* [2] that rod-shaped cells and spherical cells occurred in fluid inclusions in halite up to 26 years old from Saline Valley, although some of those variations are likely due to a naturally mixed population. Natural halite most likely contains nutrients in fluid inclusions, which would discourage starvation-survival strategies from being utilized as quickly as in nutrient-free environments of the laboratory. With time, nutrients in fluid inclusions in natural halite would deplete as microorganisms used them. The experiments in this study show that *Hbt. salinarum* and DV582A-1 favor rod-shaped cells when nutrients are available and spherically shaped cells are preferred during starvation conditions. Schubert *et al.* [4] reported that small spherical cells dominated fluid inclusion communities in 22–34 ka halite from Death Valley. Perhaps the nutrient levels in those fluid inclusions were low enough to favor spherical cells, or other factors contributed to miniaturization of cells.

### 3.4. Suitability of Hbt. salinarum and DV582A-1 for Long-Term Survival

Several observations suggest that *Hbt. salinarum* and DV582A-1 are suitable for long-term survival. Nearly all cells of these species survived 56 days of starvation. Both organisms increased cell numbers through reductive division. This survival mechanism allows cells to replicate without increasing cell volume, thus dividing the original biomass over a greater number of smaller cells [54,58]. Growth increases the survival odds of the population [58]. Only one cell needs to survive to propagate the species [50] so by increasing the size of a population, the odds of species survival increase significantly. There are also advantages to smaller cells: (1) less energy is required to perform cellular maintenance, vital to haloarchaea trapped in fluid inclusions in salt; and (2) the surface to volume ratio increases which is better for absorbing nutrients [51].

### 3.5. Comparison of Starvation-Survival Studies Using Haloarchaea

Norton and Grant [3] and Fendrihan *et al.* [43] documented the responses of haloarchaea to experimental starvation. Both studies showed starved strains of *Hbt*. *salinarum* converting from rods to cocci after being embedded in halite crystals. After entrapment, rods rounded in 2–3 weeks, but retained the rod-shape up to 3 months when the experiment concluded [3]. Fendrihan *et al.* [43] observed the conversion from rods to spheres within 1–4 days after cells were embedded in halite. However, crystallization varied anywhere from 2 to 21 days, so the conversion occurred within 3–25 days. It is unclear if the transformation took place before cells were embedded or following crystal formation.

The timeline for conversion from rods to cocci in Norton and Grant [3] and Fendrihan *et al.* [43] resembled the observations of this study in which some cells of *Hbt. salinarum* became spherical in 2–4 weeks of starvation and most others did so after 56 days. However, instead of entrapment in halite, cells in this study were stored in microcentrifuge tubes to facilitate observation of cells for the entire experimental period.

Conversion from rods to cocci was also observed by Amy and Morita [36] in 16 marine isolates but the timing varied by organism. For example, marine isolate N2 changed from rod to coccus in two months, whereas N1 took 7–9 months to change from a rod to a coccobacillus. The physical transformation from rod to spherical cells can also be caused by exposure to lower water activity, as demonstrated by Fendrihan *et al.* [59]. In that case, cellular transformations took place instantaneously when introduced to a_w_ < 0.73.

### 3.6. Implications of This Study

**Figure 10:**
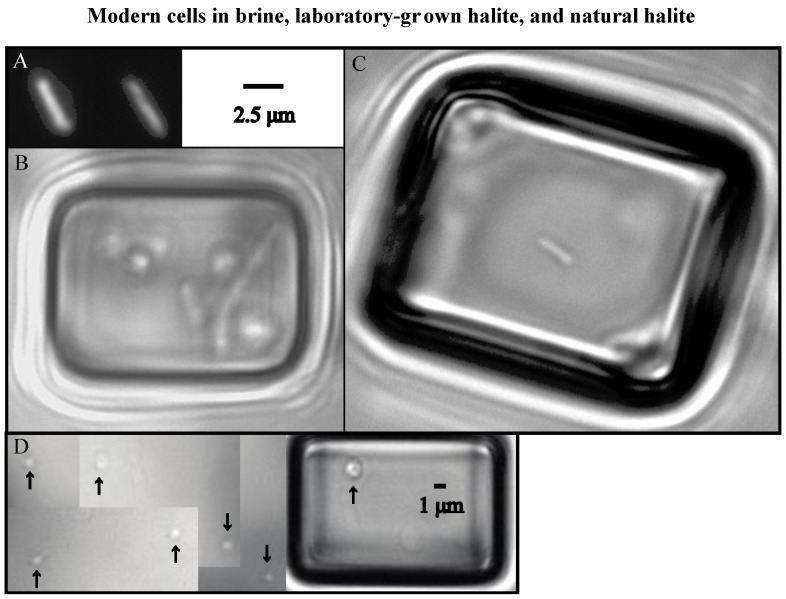
Shows haloarchaea in brine, laboratory-grown halite, and natural halite. (**A**) Two rod-shaped cells of *Hbt. salinarum* alive (treated with Live/Dead stain) after 8 weeks of nutrient-starvation; (**B**) spherical and rod-shaped cells of *Hbt. salinarum* after 3 years of starvation in fluid inclusion in laboratory-grown halite; (**C**) rod-shaped cell in fluid inclusion in natural 9 year old halite from Saline Valley, California; and (**D**) ancient spherical cells trapped in fluid inclusion in subsurface halite (depth 18.3 m) in sediment core SV-4A, Saline Valley. Arrows point to spherical cells.

The experiments described here contribute information on the timing and extent of survival mechanisms used by haloarchaea in fluid inclusions in ancient salt. Most rods converted to small spheres during experimental starvation, but not all rods made this transition (Figure 4). When cells were treated with LIVE/DEAD^®^ stain on day 56, 88% ± 6.9% of cells were found to be living, including rods (Figure 10A). It is not clear why some cells change morphology, whereas others do not. If small spherical cells are more efficient at absorbing nutrients, it would be advantageous for all rods to convert to spheres.

The evaporation of hypersaline waters allows the entrapment of microscopic communities in fluid inclusions during salt crystal formation. Two groups of microorganisms, haloarchaea and eukaryotic single-celled algae, are prevalent in fluid inclusions in halite formed in modern and ancient saline lakes [18,42]. Fluid inclusions in modern halite from Saline Valley contain haloarchaea similar in appearance to prokaryotes in modern brines, e.g., rods 1–12 µm long, cocci 0.5–2 µm, rare disks and diplococci (Figure 1). These fluid inclusions also contain *Dunaliella*, an important primary producer in hypersaline environments. *Dunaliella* produce glycerol as a compatible solute for hypersaline waters [60].

In hypersaline lakes, dense populations of haloarchaea arise following *Dunaliella* blooms [61]. As a result, it was proposed that glycerol released by *Dunaliella* from changing salt concentrations or through lysis, supplies energy for haloarchaea. This logic was applied to prokaryotes coexisting with single-celled algae in fluid inclusions in halite. When buried, halite crystals are closed systems [62]; any glycerol released by *Dunaliella* should be trapped in fluid inclusions, available to their cohabitants the haloarchaea. Since *Dunaliella* (4–20 µm in length and 2–20 µm in width, [63]) are significantly larger than haloarchaea, the amount of glycerol made by *Dunaliella* may sustain prokaryotes for millions of years [42]. However, if glycerol is available as an energy source to haloarchaea in fluid inclusions, then why are prokaryotes spherical and significantly smaller (~1 µm) in ancient fluid inclusions compared to cells in modern environments? There are many triggers for morphological changes—predation avoidance, life cycle stages, *etc.*; but the most relevant trigger in this scenario is nutrient limitation [64].

The conversion of rods to cocci is beneficial to haloarchaea under non-ideal nutrient conditions: (1) Cocci provide greater surface to volume ratios than rods, which allows nutrients to reach the cytoplasm faster [64]; (2) The size and shape of a cell impacts the rate of biochemical reactions, e.g., the time it takes for a nutrient to contact a protein in the cytoplasm; reactions are faster in a small coccus than a rod because molecules do not travel as far in a coccus [64]; (3) A small coccus requires less energy for maintenance than a rod [51]. Thus, conversion from rods to cocci should enable cells to live longer, which is essential for haloarchaea trapped in fluid inclusions in halite. However, the transformation, in itself, is an indicator of non-ideal environmental conditions.

The experiments described here used glycerol produced by *Dunaliella* as a nutrient source. The dwarfing process in microorganisms observed in natural systems [3,36,37,38,43] was successfully reproduced in this study. Rod-shaped *Hbt. salinarum* and DV582A-1 resembled prokaryotes living in modern brines and within halite. During exposure to *Dunaliella*, *Hbt. salinarum* and DV582A-1 cells became smaller; individual and paired spheres (cocci and diplococci) grew in number. When the experiments were concluded, spherical cells were close in size to haloarchaea observed in fluid inclusions in ancient halite.

The response of both genera to *Dunaliella* indicated that glycerol, alone, was not sufficient to completely satisfy the nutritional needs of either organism. If so, we would expect *Hbt. salinarum* and DV582A-1 to maintain their size and rod-shaped forms as well as conserve or increase their biomass as they did in nutrient-rich media. The responses of *Hbt. salinarum* and DV582A-1 to *Dunaliella*, however, were more similar to the behavior of these genera under starvation conditions.

Populations of *Hbt. salinarum*, in contact with *Dunaliella* cells, increased, but lost between 64% and 70% of their cell volume over 8 weeks of experimentation, similar to the 63% (population I) to 64% (population II) of cell volume lost during experimental starvation. Populations of DV582A-1 with *Dunaliella* cells increased by reductive division (days 1–14) and then decreased in concert with cell dwarfing. Dwarfing occurs when reductive division ends, *i.e.*, population size can no longer increase, but is either conserved or decreased. In dwarfing, prokaryotes consume their own endogenous material for maintenance energy; they shrink and become rounded as a result [35,51,65]. Populations of DV582A-1 plus *Dunaliella* lost 62% (populations I and II) of their cell volume compared to 65% (populations I and II) of cell volume lost during experimental starvation. DV582A-1 also experienced decreases in populations caused by cell deaths, which suggests that the population size was unstable given the amount of available nutrients, *i.e.*, glycerol. *Dunaliella* were supplied in concentrations previously documented for natural settings by Kaplan and Friedman [66] and Post [67]. Therefore it is unlikely that the number of *Dunaliella* available to DV582A-1 were insufficient for nutrient supply. DV582A-1 probably required other compounds to stabilize its population.

### 3.7. Considerations for Experimental Design in Future Work

Further research on starvation survival of haloarchaea should include chemical analyses of fluid inclusions to determine compounds available to haloarchaea. Such analyses may reveal a possible electron acceptor suitable to allow the metabolism of glycerol that was missing from the brines in these experiments. Missing compounds not provided by *Dunaliella* cells could have caused size reduction and transformation from rods to cocci in haloarchaea in these experiments, but it is unlikely to be the reason for these same changes in haloarchaea trapped in fluid inclusions. Fluid inclusions contain complex brines including compounds suitable to complete metabolic reactions because haloarchaea are metabolically versatile [68].

*Hbt. salinarum* can (1) use carbon for aerobic metabolism; (2) use bacteriorhodopsin for light-mediated ATP synthesis; and (3) ferment arginine for energy [31]. If haloarchaea in fluid inclusions in buried halite had similar capabilities to *Hbt. salinarum*, we can deduce that (1) photosynthesis was not an option due to lack of light; and (2) respiration could only be a temporary form of metabolism given a finite supply of oxygen in a fluid inclusion. Fermentation is likely the preferred form of metabolism in fluid inclusions after oxygen, with limited solubility in saline waters [56,69], is depleted through respiration. Glycerol may be consumed by some halophilic anaerobic bacteria in fluid inclusions in ancient halite through respiration or fermentation as long as nitrate is available as an electron acceptor [70,71]. Miniaturization is likely essential for survival of prokaryotes. Prokaryotes that remained large in natural fluid inclusions for at least 26 years have not miniaturized, or more likely, are probably dead. Further studies controlling environmental parameters, such as oxygen, sulfate, and amino acid availability will be crucial in determining how prokaryotes survive in salt for millennia.

## 4. Materials and Methods

### 4.1. Organisms

Two halophilic archaea were used in this study: (1) DSMZ (Deutsche Sammlung von Mikroorganismen und Zellkulturen, Braunschweig, Germany) *Halobacterium salinarum* strain 670. *Hbt. salinarum* was selected for this experiment (a) because it is easily cultured; and (b) for its ability to catabolize compounds from hypersaline environments such as glycerol and pyruvate [68]; (2) A sub-culture was grown from an ancient haloarchaeon from 34 ka halite, Death Valley salt core, designated DV582A-1 by Schubert *et al.* [4]. Its closest modern relative is *Haloterrigena thermotolerans* (98.6% similarity; Genbank FJ492049), [4]. DV582A-1 was selected for this experiment because (a) it was cultured with Pyruvate Glycerol Binghamton (PGB) medium which contained glycerol [4]; and (b) it survived within a fluid inclusion in halite for 34 ka, probably utilizing compounds, including glycerol, produced by single-celled algae resembling the modern *Dunaliella*. The response of *Hbt. salinarum* and DV582A-1 to complete starvation will be used to compare the response of these organisms with the addition of *Dunaliella*.

An axenic species of *Dunaliella* isolated from Bridger Bay, Utah was provided by Jürgen Polle, CUNY Brooklyn College.

### 4.2. Media

*Hbt. salinarum*: In a 1 liter solution, casamino acids 7.5 g, yeast extract 10.0 g, sodium citrate (C_6_H_5_Na_3_O_7_) 3.0 g, potassium chloride (KCl) 2.0 g, magnesium sulfate heptahydrate (MgSO_4_·7H_2_O) 20.0 g, iron sulfate heptahydrate (FeSO_4_·7H_2_O) 0.05 g, manganese sulfate monohydrate (MnSO_4_·H_2_O) 0.20 mg, and sodium chloride (NaCl) 250.0 g were added. The pH was adjusted to 7.4.

Ancient strain DV582A-1: Dipotassium phosphate (K_2_HPO_4_) 0.5 g, magnesium sulfate heptahydrate (MgSO_4_·7H_2_O) 2.0 g, potassium chloride (KCl) 4.0 g, glycerol 2.5 g, sodium pyruvate (C_3_H_3_NaO_3_) 2.5 g, ammonium sulfate ((NH_4_)_2_SO_4_) 1.0 g, and sodium chloride (NaCl) 250.0 g were combined in 1 L solution.

*Dunaliella*: This recipe was modified from Pick *et al.* [72]. In a 1 liter solution, combine 600 mL of H_2_O, 250 mL of 4 M sodium chloride (NaCl), 100 mL of concentrated mix, and 1 mL of micronutrients. At room temperature 50 mL of 0.5 M sodium bicarbonate (NaHCO_3_) was added. In a 1 L solution, the concentrated mix contains 450 mL of H_2_O, 200 mL of 2 M Tris-HCl (pH 7.5), 50 mL of 1 M potassium nitrate (KNO_3_), 50 mL of 1M magnesium sulfate (MgSO_4_), 50 mL of 60 mM calcium chloride (CaCl), 100 mL of 20 mM monopotassium phosphate (KH_2_PO_4_), and 100 mL of 0.4 mM iron (III) chloride (FeCl_3_) in 4 mM EDTA (pH 7.5). Micronutrients contain 150 mM boric acid (H_3_BO_3_), 10 mM manganese (II) chloride (MnCl_2_), 0.8 mM zinc chloride (ZnCl_2_), 0.3 mM copper (II) chloride (CuCl_2_), 2 mM sodium molybdate (Na_2_MoO_4_), 2mM sodium metavanadate (NaVO_3_), and 0.2 mM cobalt (II) chloride (CoCl_2_).

All media and glassware were autoclaved at 121 °C (17 psi) for 30 min. Inoculation of media and extraction of subsamples took place under sterile conditions created by a class IIA Baker-made SterilGARD III laminar flow hood with an HEPA filter equipped with a germicidal UV light.

### 4.3. Experimental Details

Haloarchaea: Cultures were incubated at 37 °C. Two, 1 mL aliquots of each stock culture were distributed in separate microcentrifuge tubes. *Hbt. salinarum* and DV582A-1 were collected from the late exponential phase/early stationary phase of their growth cycles. Complete nutrient starvation was achieved by pelletizing cells at 8000 rpm for 90 seconds in an Eppendorf *miniSpin* microcentrifuge, discarding the supernatant, and resuspending the pellet with 1 mL of 4M NaCl sterile carbon-free brine. This washing procedure was repeated five times in order to remove all growth media. The washing protocol was tested with LIVE/DEAD^®^ *Bac*Light™ stain from Invitrogen (Carlsbad, CA, USA) and was determined not to impact physical cellular characteristics or population viability. Washed cells were diluted or concentrated to achieve natural population densities of haloarchaea found in modern brines (2.3 × 10^6^–9 × 10^6^ cells/mL, [66], to 2 × 10^7^–2 × 10^8^ cells/mL, [73]). Each tube was gently mixed with the Maxi Mix II vortex to distribute cells evenly, after which they were stored in the dark.

*Dunaliella*: Cultures were incubated at 37 °C with direct natural sunlight. Cells were harvested when the population reached a density similar to that found in the environment, 4 × 10^4^ cells/mL [66] to 2 × 10^5^ cells/mL [67]. The growth medium was rinsed from *Dunaliella* once with a sterile sodium chloride solution so that haloarchaea had no alternative nutrient sources besides the algae. Cells were gently pelletized, the supernatant discarded, and the pellet resuspended in brine. Microscopic observations of rinsed *Dunaliella* showed that some cells were intact while others were lysed. Lysis occurred during resuspension and therefore, any glycerol that leaked from cells was still present in the brine solution along with the algae. This solution was added to starved populations of *Hbt. salinarum* and DV582A-1.

Population size and cellular lengths were recorded at regular time intervals in both nutrient-rich media, starved cultures, and nutrient-controlled conditions (lysed *Dunaliella*) using the Petroff-Hausser Counting Chamber on a Zeiss Axio Imager. A1 compound light microscope with a 100× oil immersion objective (PLAN APO 100×/1.4 oil). These time intervals were chosen because the generation time of haloarchaea is relatively slow, on the order of hours to days. Cells were monitored more frequently at the beginning of each experiment to observe how they acclimated to new nutrient conditions. When it was determined that their morphological transformations were occurring gradually, monitoring became less frequent. Cells were counted in three different fields and the population was averaged. The standard deviation of the population size represents the variation in cell counts found within these three fields on each monitored day. Average cell length was calculated by measuring 100 cells from photographs, taken with an AxioCam MRm black and white camera, and analyzed with AxioVision software (version 4.8.2.0, Carl Zeiss, Jena, Germany). The standard deviation of average cell lengths shows the variation in the 100 cells measured on each monitored day. Monitoring continued until no further morphological changes were observed, *i.e.*, when cells approached 1 μm in diameter and the majority of each population converted to spheres. LIVE/DEAD^®^ *Bac*Light™ stain (Invitrogen, Waltham, MA, USA) was applied at the end of each experiment to estimate culture viability.

### 4.4. Important Definitions

Growth: Refers to an increase in cell number or population size [21]. It can occur by binary fission or reductive cell division.

Binary fission: Binary fission happens when a cell doubles its minimum size in the presence of nutrients and divides [21,52].

Reductive cell division: This type of cell division occurs in stationary phase if a cell replicates its DNA prior to nutrient expiration, during exponential growth [51]. It increases population size; however cells do not gain biomass. It is also known as fragmentation [52,53,54].

Miniaturization: Here it refers to a reduction in cell size caused by self-digestion of endogenous material. It is instigated by starvation and only occurs after reductive cell division has ended. It is commonly referred to as dwarfing [35,51,65] or ultramicrocell formation [34]. It is important to distinguish the process of miniaturization (dwarfing) as it is used here, from the “miniaturization” used by Amy and Morita [36] and Schubert *et al.* [2] that describes a general decrease in cell size in response to nutrient limitation.

## 5. Conclusions

The results of this study show that *Hbt. salinarum* and DV582A-1 are well suited and organized for extended starvation. They responded to starvation rapidly; their physical appearance began changing after several days. After 56 days, the size and shape of most starved cells began to resemble those in the fluid inclusions of ancient salt deposits. This timeline agrees with previous examinations of haloarchaea in nutrient-free environments by Norton and Grant [3], and Fendrihan *et al.* [43]. *Hbt. salinarum* and DV582A-1 reduced cell lengths and most rods transformed into spheres within 56 days, even in the presence of glycerol, a nutrient produced by *Dunaliella*. These results resembled the organism response to starvation conditions and are consistent with observations of microorganism communities of prokaryotes and *Dunaliella*-like algae in fluid inclusions in ancient halite. 
